# Residual clinical damage after COVID-19: A retrospective and prospective observational cohort study

**DOI:** 10.1371/journal.pone.0239570

**Published:** 2020-10-14

**Authors:** Rebecca De Lorenzo, Caterina Conte, Chiara Lanzani, Francesco Benedetti, Luisa Roveri, Mario G. Mazza, Elena Brioni, Giacomo Giacalone, Valentina Canti, Valentina Sofia, Marta D’Amico, Davide Di Napoli, Alberto Ambrosio, Paolo Scarpellini, Antonella Castagna, Giovanni Landoni, Alberto Zangrillo, Emanuele Bosi, Moreno Tresoldi, Fabio Ciceri, Patrizia Rovere-Querini

**Affiliations:** 1 School of Medicine, Vita-Salute San Raffaele University, Milan, Italy; 2 Division of Immunology, Transplantation and Infectious Diseases, IRCCS San Raffaele Scientific Institute, Milan, Italy; 3 Unit of Nephrology, IRCCS San Raffaele Scientific Institute, Milan, Italy; 4 Unit of Psychiatry and Clinical Psychobiology, Division of Neuroscience, IRCCS San Raffaele Scientific Institute, Milan, Italy; 5 Department of Neuroscience, INSPE, IRCCS San Raffaele Scientific Institute, Milan, Italy; 6 Clinical Governance Division, IRCCS San Raffaele Scientific Institute, Milan, Italy; 7 Department of Anaesthesia and Intensive Care, IRCCS San Raffaele Scientific Institute, Milan, Italy; 8 Unit of General Medicine and Advanced Care, IRCCS San Raffaele Scientific Institute, Milan, Italy; BronxCare Health System, Affiliated with Icahn School of Medicine at Mount Sinai, UNITED STATES

## Abstract

Data on residual clinical damage after Coronavirus disease-2019 (COVID-19) are lacking. The aims of this study were to investigate whether COVID-19 leaves behind residual dysfunction, and identify patients who might benefit from post-discharge monitoring. All patients aged ≥18 years admitted to the Emergency Department (ED) for COVID-19, and evaluated at post-discharge follow-up between 7 April and 7 May, 2020, were enrolled. Primary outcome was *need of follow-up*, defined as the presence at follow-up of at least one among: respiratory rate (RR) >20 breaths/min, uncontrolled blood pressure (BP) requiring therapeutic change, moderate to very severe dyspnoea, malnutrition, or new-onset cognitive impairment, according to validated scores. Post-traumatic stress disorder (PTSD) served as secondary outcome. 185 patients were included. Median [interquartile range] time from hospital discharge to follow-up was 23 [20–29] days. 109 (58.9%) patients needed follow-up. At follow-up evaluation, 58 (31.3%) patients were dyspnoeic, 41 (22.2%) tachypnoeic, 10 (5.4%) malnourished, 106 (57.3%) at risk for malnutrition. Forty (21.6%) patients had uncontrolled BP requiring therapeutic change, and 47 (25.4%) new-onset cognitive impairment. PTSD was observed in 41 (22.2%) patients. At regression tree analysis, the ratio of arterial oxygen partial pressure to fractional inspired oxygen (PaO_2_/FiO_2_) and body mass index (BMI) at ED presentation, and age emerged as independent predictors of the *need of follow-up*. Patients with PaO_2_/FiO_2_ <324 and BMI ≥33 Kg/m^2^ had the highest odds to require follow-up. Among hospitalised patients, age ≥63 years, or age <63 plus non-invasive ventilation or diabetes identified those with the highest probability to need follow-up. PTSD was independently predicted by female gender and hospitalisation, the latter being protective (odds ratio, OR, 4.03, 95% confidence interval, CI, 1.76 to 9.47, p 0.0011; OR 0.37, 95% CI 0.14 to 0.92, p 0.033, respectively). COVID-19 leaves behind physical and psychological dysfunctions. Follow-up programmes should be implemented for selected patients.

## Introduction

Since the identification of Severe Acute Respiratory Syndrome-Coronavirus-2 (SARS-CoV-2) as the causative agent of Coronavirus disease-2019 (COVID-19), more than four million cases were reported worldwide, mortality reaching 6.68% as of the 20^th^ of May 2020 [1]. The majority of affected patients manage to overcome the acute phase of the disease and appear to achieve clinical recovery [2]. Knowledge of early disease characteristics accumulates rapidly. However, *sequelae* of COVID-19 remain unexplored. It seems, therefore, reasonable to question whether it is safe to lower the guard. Monitoring recovered patients over time might be revelatory of what comes next, maximizing preparedness and optimizing medical care.

Persistent radiological lung abnormalities and breathing difficulties were reported in patients recovered from previous coronavirus diseases [3, 4]. The applicability of these observations to SARS-CoV-2-infected patients is unknown [5, 6]. Inflammation is a recognized promoter of tissue fibrosis [7]. As such, the burden of pulmonary dysfunction after COVID-19 recovery may be substantial. The suggested neurotropism of SARS-CoV-2 might entail neurocognitive *sequelae* of COVID-19 [8], and the persistence of other disease features cannot be excluded [9, 10]. Psychological health in convalescent patients is also a matter of concern. Fear of infection-associated complications, prohibition of human contact, and uncertainty about reacceptance in society may jeopardize mental well-being and influence quality of life, prompting to the need of adequate mental counselling.

Alertness and awareness of what to expect are crucial not to underestimate health problems and to guarantee timely interventions. This would aid in preventing national health care systems from being overwhelmed by the sudden surge of conditions requiring medical assistance.

With the belief that hospital discharge is far from being the endpoint of monitoring and precautionary measures, we set up a COVID-19 follow-up outpatient clinic to longitudinally follow patients recovered from COVID-19. Here, we report a first assessment of the information gathered on COVID-19 *sequelae* and propose strategies to identify patients who may benefit from continued monitoring.

## Methods

### Design and study population

This is a retrospective and prospective cohort study included in an extensive monocentric observational investigation, the COVID-BioB study, implemented at San Raffaele University Hospital in Milan, Italy. All patients aged 18 years or older, admitted to San Raffaele University Hospital from 25 February 2020 with confirmed SARS-CoV-2 infection were consecutively enrolled in the COVID-BioB study. Confirmed infection was defined as positive real-time reverse-transcriptase polymerase chain reaction (RT-PCR) from a nasopharyngeal and/or throat swab. Patients with clinical and radiological findings suggestive of COVID-19 pneumonia were selected for follow-up evaluation after hospital discharge at the COVID-19 Follow-up Outpatient Clinic of San Raffaele University Hospital. Patients evaluated since the start of the Clinic (7 April 2020) up to 7 May 2020 were included for the present analysis. Patients admitted for reasons other than COVID-19 who subsequently tested positive for SARS-CoV-2 at routine screening were excluded.

The COVID-BioB study protocol conforms to the declaration of Helsinki, was approved by the Hospital Ethics Committee, namely *Comitato Etico Ospedale San Raffaele* (*CE-OSR*, protocol no. 34/int/2020), and registered on ClinicalTrials.gov (NCT04318366). For patients able to provide a signed informed consent (IC) at the time of hospital admission, written IC was obtained prior to data collection. Otherwise, patients were consented as soon as they were able to sign. This study is reported in compliance with the STROBE statement [11].

### Follow-up evaluation

**Fig 1:**
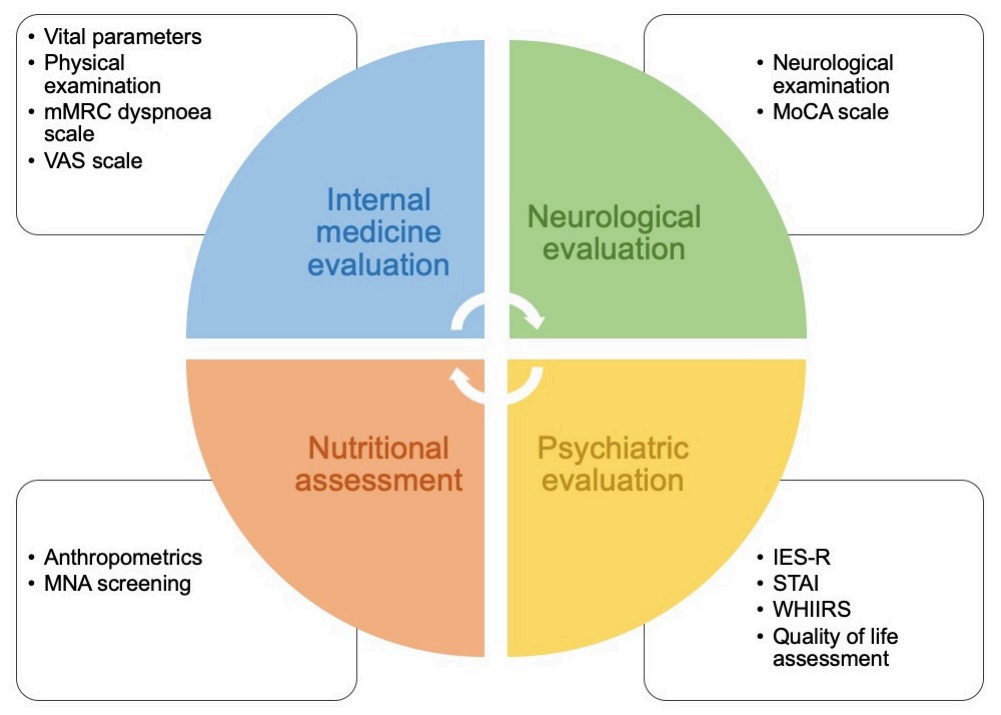
Multidisciplinary organisation and assessment measures of the COVID-19 Follow-up Outpatient Clinic at San Raffaele University Hospital. mMRC, modified Medical Research Council. VAS, visuo-analog scale. MoCA, Montreal Cognitive Assessment. MNA, Mini Nutritional Assessment. IES-R, Impact of Event Scale-Revised. STAI-Y, State-Trait Anxiety Inventory form Y. WHIIRS, Women’s Health Initiative Insomnia Rating Scale.

A comprehensive evaluation of physical, neurological, cognitive and mental health was performed by a multidisciplinary team consisting of internists, nutritionists, neurologists, and psychiatrists (Fig 1). Data about the initial presentation of COVID-19 and the disease course were retrospectively scrutinized from medical records in the presence of the patient during follow-up evaluation and collected. Complete physical examination and vital sign assessment were integrated with detailed patient medical history. The modified Medical Research Council (mMRC) scale for dyspnoea was used to quantify residual shortness of breath [12], and a visuo-analog scale (VAS) for self-rated health status [13]. Percent of body weight change and the Mini Nutritional Assessment (MNA) screening tool served as indicators of nutritional status [14]. The MNA screening tool was initially developed for detecting undernutrition in the elderly, but it has subsequently been adopted in several clinical settings and patient populations [15–17]. The tool identifies individuals at risk of malnutrition or malnourished based on the presence of reduction in food intake (due to loss of appetite, digestive problems, chewing or swallowing difficulties), disease burden (psychological stress or acute disease in the previous 3 months or presence of neuropsychological problems), weight loss, body mass index or reduced mobility. An MNA value ≤7 indicates malnutrition and a score between 8 and 11 identifies patients at risk of malnutrition [14]. Complete neurological examination was performed to investigate neurological *sequelae*. Cognitive function was assessed through the Montreal Cognitive Assessment (MoCA) score [18], and cognitive impairment was defined by a score <24 in the absence of known history of neurocognitive disease.

Psychiatric unstructured clinical interview was conducted to investigate the presence of a current major psychiatric disorder (depressive disorders, bipolar and related disorders, anxiety disorders, psychotic disorders, eating disorders, and trauma-related disorders) according to the Diagnostic and Statistical Manual of Mental Disorders (DSM-5). Validated self-report questionnaires were used to assess quality of life through the World Health Organization Quality of Life (WHOQOL-BREF), post-traumatic stress disorder (PTSD) through the Impact of Events Scale-Revised (IES-R), anxiety through the State-Trait Anxiety Inventory form Y (STAI-Y), and insomnia through the Women’s Health Initiative Insomnia Rating Scale (WHIIRS) [19–22].

### Variables

Demographical data (i.e. age, gender, and ethnicity), comorbidities (i.e. hypertension, HTN, coronary artery disease, CAD, diabetes mellitus, DM, chronic obstructive pulmonary disease, COPD, chronic kidney disease, CKD, active cancer, and current psychiatric disorder according to DSM-5), as well as body mass index (BMI), axillary body temperature, and laboratory values (i.e. the ratio of arterial oxygen partial pressure, PaO_2_ in mmHg, to fractional inspired oxygen, FiO_2_, expressed as a fraction, PaO_2_/FiO_2_, white blood cell count, WBC, neutrophil to lymphocyte ratio, NLR, liver enzymes, lactate dehydrogenase, LDH, C-reactive protein, CRP, estimated glomerular filtration rate, eGFR using the CKD-EPI equation) at ED presentation were extracted for all patients. Acute respiratory distress syndrome (ARDS) was defined as PaO_2_/FiO_2_ <300 [23]. For hospitalised patients, length of stay (LoS), transfer to the intensive care unit (ICU), and non-invasive ventilation (NIV) administration were also recorded.

Data collected at the follow-up visit included vital parameters, percent of body weight change from hospital admission, mMRC for dyspnoea, MNA, VAS and WHOQOL scores, and the presence of cognitive impairment, PTSD, anxiety, and insomnia according to the generally accepted cut-off scores (MoCA <24, IES-R ≥33, STAI-state ≥40, STAI-trait ≥40, and WHIIRS ≥9, respectively). Previous need for psychiatric interventions and previous or current intake of psychotropic drugs were also collected. Tachypnoea was defined as respiratory rate (RR) >20 breaths/min [24], measured by counting respiratory chest movements of over a period of 60 seconds.

Prior to analysis, data were cross-checked with medical charts and verified by data managers and clinicians for accuracy.

### Outcomes

To investigate the relevance of the follow-up visit, we created a composite dichotomous variable, i.e. *need of follow-up*, which identified patients requiring medical advice after COVID-19 recovery. Accordingly, the *need of follow-up* was defined by the presence of at least one of the following: *i)* tachypnoea, *ii)* mMRC for dyspnoea score ≥2, *iii)* uncontrolled blood pressure requiring a change in therapy (increase in dose or new prescription of at least one anti-hypertensive drug, i.e. diuretics, calcium channel blockers, angiotensin-converting enzyme inhibitors, angiotensin receptor blockers, and beta blockers), *iv)* MNA score ≤7, *v)* presence of cognitive impairment. The *need of follow-up* variable represented the primary outcome. Psychiatric disturbances were not included in the primary outcome for the purpose of the analysis, and PTSD was used as secondary outcome.

### Statistical analyses

Descriptive statistical analyses were performed for all variables. Dichotomous variables were expressed as absolute frequencies (percentage), and continuous variables as medians [IQR]. Group comparisons were performed using the χ2 test or Fisher’s exact test for categorical variables, and the Mann-Whitney U test for continuous variables.

To investigate the impact of individual variables on the *need of follow-up*, we performed univariable and multivariable logistic regression analyses both for the entire cohort and for hospitalised patients only. We subsequently employed a regression tree (RT) algorithm to identify risk groups based on the *need of follow-up*, within the entire cohort (RT 1) and the hospitalised population (RT 2). The RT algorithm uses recursive partitioning to sequentially split a cluster of patients into increasingly homogeneous sub-groups based on several independent variables, selecting the optimal sequence of classifications as defined by a hierarchy of prognostic factors and associated cut-points [25]. Demographical data, comorbidities, BMI, clinical and laboratory features at ED presentation, and hospitalisation due to COVID-19 were included as predictors in RT 1. LoS, NIV administration, and transfer to ICU, together with demographical data and comorbidities, were used as covariates in RT 2. The results of these analyses were graphically represented. The area under the receiver operating characteristic (ROC) curve (ROC_AUC_) was used as a quality metric of the regression trees.

Univariable and multivariable logistic regression analyses were employed to identify predictors of PTSD among age, gender, BMI at ED presentation, comorbidities, hospitalisation, and ARDS.

Missing data was not imputed.

All statistical analyses were performed using R statistical package (version 4.0.0, R Foundation for Statistical Computing, Vienna, Austria), with a two-sided significance level set at p <0.05.

### Patient and public involvement

As the study addresses an urgent unmet clinical need in response to a global public health emergency, patients and members of the public were not directly involved in the design, conduct, or reporting of this research.

## Results

### Patient characteristics at baseline and at follow-up

From 7 April to 7 May 2020, a total of 195 COVID-19 patients were evaluated at the COVID-19 Follow-up Outpatient Clinic of San Raffaele University Hospital. Of these, 10 had been admitted for reasons other than COVID-19 and were therefore excluded for the present analysis. All patients included (n = 185) had a positive SARS-CoV-2 RT-PCR test result from a nasopharyngeal and/or throat swab. Characteristics at disease onset and follow-up assessment measures of the cohort are reported in Tables 1 and 2, respectively. Of the 185 patients included in the analysis, 68.1% had been hospitalised, while the rest were discharged from the ED. Most inpatients received hydroxychloroquine in conjunction with lopinavir/ritonavir, which was the standard therapy for COVID-19 at our Institution at the time patients included in the study were admitted to hospital. Additional treatments were prescribed based on the severity of the clinical picture. Patients managed at home were prescribed symptomatic treatments. Patients were assessed after a median [interquartile range, IQR] time from hospital discharge of 23 [20–29] days.

**Table 1:** General features of COVID-19 patients.

Variable	All Cohort	Discharged from ED	Hospitalised	P
**No. of patients**	185 (100)	59 (31.9)	126 (68.1)	
**Age (years)**	57 (48; 67)	50 (40.5; 57.7)	61 (51.2; 69)	<0.0001
**Female gender**	62 (33.5)	28 (47.5)	34 (27)	0.0098
**Ethnicity**				0.029
** European (Eastern/Western)**	168 (90.8)	49 (83.1)	119 (94.4)	
** Hispanic (South America)**	16 (8.6)	9 (15.3)	7 (5.6)	
** African-American (Black)**	1 (0.5)	1 (1.7)	0 (0)	
**BMI (Kg/m**^**2**^**)**	27.2 (24.7; 30.5)	26.2 (24.1; 30.7)	27.8 (25.4; 30.5)	0.12
***Comorbidities***				
** HTN**	70 (37.8)	15 (25.4)	55 (43.7)	0.029
** CAD**	12 (6.5)	5 (8.5)	7 (5.6)	0.65
** DM**	21 (11.4)	4 (6.8)	17 (13.5)	0.28
** COPD**	2 (1.1)	0 (0)	2 (1.6)	0.84
** CKD**	3 (1.6)	0 (0)	3 (2.4)	0.58
** Active cancer**	3 (1.6)	1 (1.7)	2 (1.6)	1.00
** Psychiatric disorder**	40 (21.6)	15 (25.4)	25 (19.8)	0.77
***At ED presentation***				
** PaO**_**2**_**/FiO**_**2**_	314.8 (263.8; 357.6)	371.4 (333.8; 409.5)	296.4 (249.6; 330.7)	<0.0001
** Body temperature (°C)**	37.8 (37; 38.3)	37.5 (36.6; 38)	37.9 (37.1; 38.5)	0.0088
** WBC (x10**^**9**^**/L)**	6 (4.8; 7.9)	5.5 (4.2; 6.6)	6.3 (4.9; 8.3)	0.017
** NLR**	3.5 (2; 6.3)	2.7 (1.9; 3.9)	4.4 (2.2; 7.3)	0.0018
** AST (U/L)**	39 (27; 55.2)	27 (24; 47)	44 (32; 58.5)	<0.0001
** ALT (U/L)**	35 (22.8; 55)	27 (21; 48)	38 (24; 58)	0.058
** LDH (U/L)**	330 (248; 409.5)	249 (208; 340)	362.5 (281.2; 426)	<0.0001
** CRP (mg/dL)**	46.7 (14.8; 100.8)	12.3 (4.9; 48.8)	67.2 (29; 120.2)	<0.0001
**eGFR (mL/min)**	85.5 (68.3; 97.2)	92.8 (76.2; 105.8)	79.8 (66.4; 92.4)	0.0001
**LoS**	9.5 (6; 15)	-	9.5 (6; 15)	-
**Transfer to ICU**	4 (2.2)	-	4 (3.2)	-
**NIV**	32 (17.3)	-	32 (25.4)	-

Dichotomous variables were expressed as count (percentage) and continuous variables as median (interquartile range).

Abbreviations: BMI, body mass index; ED, Emergency Department; PaO_2_/FiO_2_, arterial oxygen partial pressure/fractional inspired oxygen; WBC, white blood cell count; NLR, neutrophil to lymphocyte ratio; AST, aspartate aminotransferase; ALT, alanine aminotransferase; LDH, lactic dehydrogenase; CRP, C-reactive protein; eGFR, estimated glomerular filtration rate; HTN, arterial hypertension; CAD, coronary artery disease; DM, diabetes mellitus; COPD, chronic obstructive pulmonary disease; CKD, chronic kidney disease; LoS, length of stay; ICU, intensive care unit; NIV, non-invasive ventilation.

**Table 2:** Features at follow-up of COVID-19 patients.

Variable	All Cohort	Discharged from ED	Hospitalised	P
**Time from hospital discharge to follow-up visit (days)**	23 (20; 29)	26 (22; 33.5)	21.5 (19; 26.8)	0.00011
**SBP (mmHg)**	132.5 (123; 144.8)	130 (120; 141)	134 (125; 145)	0.17
**DBP (mmHg)**	78 (70; 85)	75 (70; 86)	80 (70; 85)	0.99
**HR (bpm/min)**	79 (70; 90)	80 (70.2; 91)	78 (70; 88)	0.43
**RR (breaths/min)**	16 (14; 20)	16 (14; 18)	18 (15; 20)	0.052
**SpO**_**2**_ **(%)**	98 (97; 99)	98.5 (97.2; 99)	98 (97; 99)	0.016
**Weight change (%)**	-1.3 (-5; 1.4)	0 (-3.3; 2.1)	-2 (-5; 1.2)	0.074
**Risk of malnutrition**	106 (57.3)	25 (42.4)	81 (64.3)	0.008
**Malnutrition**	10 (5.4)	5 (8.5)	5 (4)	0.36
**mMNA score**	11 (9–12)	11 (8.5–12.5)	11 (9–12)	0.88
**mMRC for dyspnoea scale**				0.72
** Mild dyspnoea**	42 (22.7)	11 (18.6)	31 (24.6)	
** Moderate dyspnoea**	8 (4.3)	4 (6.8)	4 (3.2)	
** Severe dyspnoea**	5 (2.7)	2 (3.4)	3 (2.4)	
** Very severe dyspnoea**	3 (1.6)	1 (1.7)	2 (1.6)	
**Tachypnoea**	41 (22.2)	8 (13.6)	33 (26.2)	0.051
**Uncontrolled BP requiring therapeutic change**	40 (21.6)	14 (23.7)	26 (20.6)	0.78
**WHOQOL**				
** Total**	99 (90; 107.2)	93 (85; 105.5)	100 (92; 109)	0.081
** Physical health/Level of independence**	16 (13.7; 17.1)	14.9 (13.4; 17.1)	16 (13.7; 17.1)	0.47
** Psychological**	14.7 (13.3; 16.7)	13.3 (12; 16)	15.3 (13.3; 16.7)	0.0084
** Social relations**	16 (14.7; 17.3)	16 (13.3; 17.3)	16 (14.7; 17.3)	0.42
** Environment**	15 (13.5; 17)	14 (13; 16.5)	15 (13.5; 17)	0.32
**VAS pain**	77.5 (75; 90)	75 (75; 90)	80 (75; 95)	0.68
**Cognitive impairment**	47 (25.4)	11 (18.6)	36 (28.6)	0.26
**Insomnia**	51 (27.6)	20 (33.9)	31 (24.6)	0.32
**Anxiety**	55 (29.7)	23 (39)	32 (25.4)	0.046
**PTSD**	41 (22.2)	23 (39)	18 (14.3)	0.00054

Dichotomous variables were expressed as count (percentage) and continuous variables as median (interquartile range).

Abbreviations: SBP, systolic blood pressure; DBP, diastolic blood pressure; HR, heart rate; RR, respiratory rate; SpO_2_, peripheral oxygen saturation; mMNA, mini nutritional assessment; mMRC, modified Medical Research Council; WHOQOL, World Health Organization quality of life; VAS, visuo-analog scale; PTSD, post-traumatic stress disorder.

Hospitalised patients were older than patients discharged from the ED, more commonly males and white. The two populations did not differ in terms of BMI at hospital admission and medical history, with the exception of HTN, which was more frequent in hospitalised patients. Laboratory findings at ED presentation of hospitalised and non-hospitalised patients are presented in Table 1.

At follow-up evaluation, 54 (29.2%) patients had shortness of breath or were tachypnoeic. 116 (62.7%) patients were malnourished or at risk for malnutrition, and approximately one quarter of patients achieved MoCA scores compatible with cognitive impairment, despite no known history of cognitive disorders. Psychiatric disturbances including anxiety, insomnia, or PTSD were observed in 83 (44.9%) patients (Table 2).

Hospitalised patients had a tendency towards a more important weight loss during disease and towards higher RR values, compared with patients discharged from the ED. Conversely, patients discharged from the ED had lower WHOQOL scores, reflecting a decreased quality of life, especially in the psychological domain. Anxiety and PTSD were more frequent among patients discharged from the ED (Table 2).

### Need of follow-up

**Fig 2:**
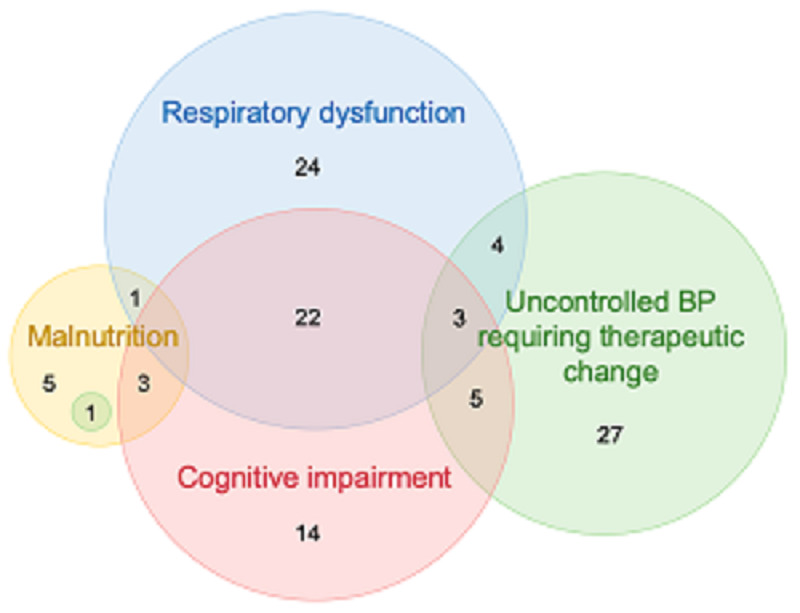
Prevalence of the main follow-up assessment measures. Respiratory dysfunction was defined as respiratory rate >20 breaths/min or modified Medical Council Research scale for dyspnoea ≥2. Depicted numbers indicate the absolute counts of patients in each set. One malnourished patient had uncontrolled blood pressure levels requiring a change in therapy. BP, blood pressure.

The *need of follow-up*, defined as the presence at follow-up evaluation of at least one among RR >20 breaths/min, uncontrolled blood pressure requiring therapeutic change, moderate to very severe dyspnoea, malnutrition, or new-onset cognitive impairment, was present in 109 (58.9%) patients (Fig 2). This number rose to 126 (68.1%) when including PTSD. No significant difference in the *need of follow-up* was found between hospitalised patients (75 of 126, 59.5%) and patients discharged from the ED (34 of 59, 57.6%). Age predicted the *need of follow-up* at regression analyses in the entire cohort. Specifically, for each additional year of age, the odds of requiring post-discharge monitoring increased by 4% (Table 3). Univariable and multivariable regression analyses predicting the *need of follow-up* within the hospitalised population are described in Table 4.

**Table 3:** Univariable and multivariable logistic regression analyses predicting the *need of follow-up* within the entire cohort (n = 185).

	Univariate			Multivariate		
	OR	95% CI	P	OR	95% CI	P
**Age (years)**	1.040	1.013 to 1.061	0.0031	1.033	1.003 to 1.067	0.037
**Female gender**	1.76	0.94 to 3.37	0.085			
**Ethnicity**	1.42	0.61 to 3.95	0.45			
**BMI (Kg/m**^**2**^**)**	1.033	0.97 to 1.10	0.30			
***Comorbidities***						
** HTN**	1.18	0.64 to 2.17	0.60			
** CAD**	0.68	0.20 to 2.25	0.51			
** DM**	1.15	0.46 to 3.04	0.78			
** CKD**	1.41	0.13 to 30.66	0.78			
** Active cancer**	0.35	0.016 to 3.67	0.39			
** Psychiatric disorder**	1.27	0.62 to 2.70	0.52			
***At ED presentation***						
** PaO**_**2**_**/FiO**_**2**_	0.99	0.98 to 0.99	0.002	0.99	0.99 to 0.99	0.054
** Body temperature (°C)**	1.12	0.81 to 1.55	0.50			
** WBC**	0.96	0.87 to 1.07	0.48			
** NLR**	1.026	0.96 to 1.011	0.48			
** AST**	1.00	0.99 to 1.01	0.73			
** ALT**	1.00	0.99 to 1.01	0.57			
** LDH**	1.00	1.0004 to 1.01	0.030	1.00	0.99 to 1.00	0.95
** PCR**	1.00	0.99 to 1.01	0.19			
** eGFR**	0.99	0.97 to 1.00	0.11			
**Hospitalisation**	1.081	0.57 to 2.02	0.81			

Abbreviations: OR, odds ratio; CI, confidence interval; BMI, body mass index; HTN, arterial hypertension; CAD, coronary artery disease; DM, diabetes mellitus; CKD, chronic kidney disease; ED, Emergency Department; PaO_2_/FiO_2_, ratio of arterial oxygen partial pressure to fractional inspired oxygen; WBC, white blood cell count; NLR, neutrophil to lymphocyte ratio; AST, aspartate aminotransferase; ALT, alanine aminotransferase; LDH, lactic dehydrogenase; CRP, C-reactive protein; eGFR, estimated glomerular filtration rate.

**Table 4:** Univariable and multivariable logistic regression analyses predicting the *need of follow-up* within the hospitalised cohort (n = 126).

	Univariate			Multivariate		
	OR	95% CI	P	OR	95% CI	P
**Age (years)**	1.043	1.01 to 1.978	0.012	1.038	1.00 to 1.08	0.055
**Female gender**	1.61	0.71 to 3.79	0.26			
**Ethnicity**	0.90	0.19 to 4.74	0.90			
***Comorbidities***						
** HTN**	1.51	0.73 to 3.15	0.27			
** CAD**	0.88	0.19 to 4.65	0.87			
** DM**	1.26	0.45 to 3.89	0.67			
** CKD**	1.34	0.13 to 29.35	0.81			
**Psychiatric disorder**	1.25	0.50 to 3.27	0.64			
**NIV**	2.064	0.89 to 5.15	0.10			
**Transfer to ICU**	0.67	0.078 to 5.75	0.70			
**LoS**	1.017	0.97 to 1.074	0.53			

Abbreviations: OR, odds ratio; CI, confidence interval; HTN, arterial hypertension; CAD, coronary artery disease; DM, diabetes mellitus; CKD, chronic kidney disease; NIV, non-invasive ventilation; ICU, intensive care unit; LoS, length of stay.

**Fig 3:**
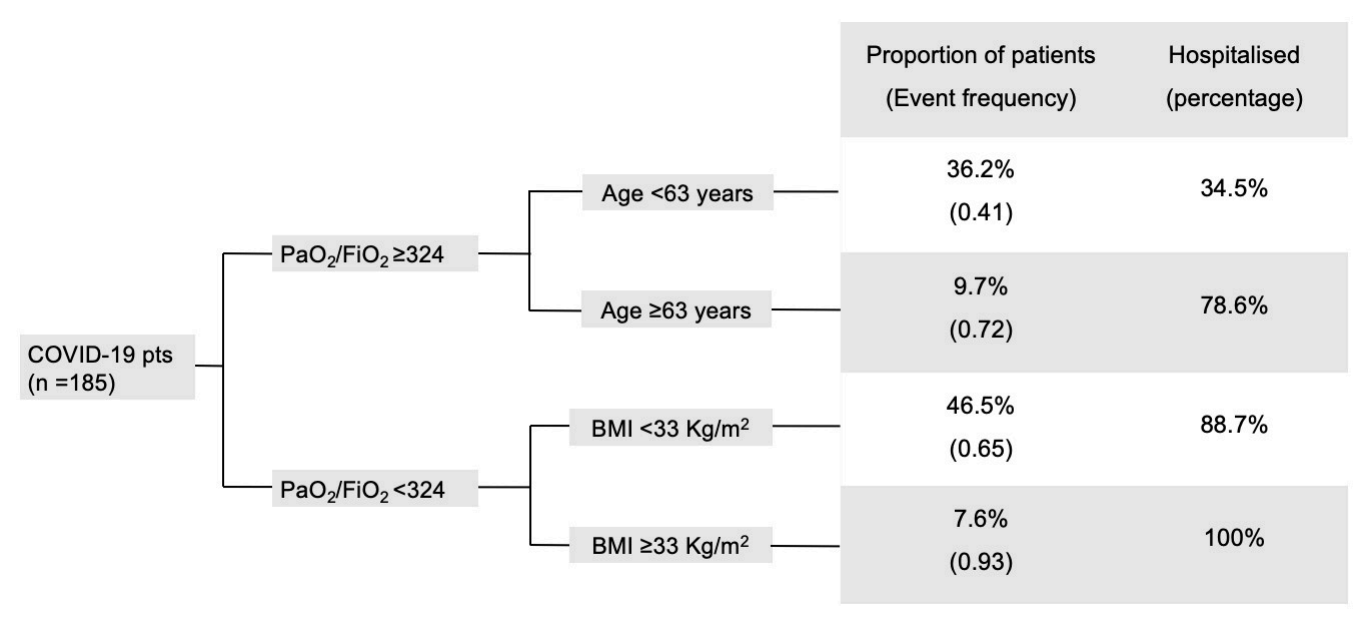
Regression tree analysis (RT 1) to predict the need of post-discharge follow-up among patients recovered from COVID-19. Event frequency defines the proportion of patients needing follow-up. The prevalence of hospitalisation in the obtained groups is depicted. Age, gender, ethnicity, history of hypertension, coronary artery disease, chronic kidney disease, diabetes mellitus, body mass index (BMI), axillary body temperature, ratio of arterial oxygen partial pressure to fractional inspired oxygen (PaO_2_/FiO_2_), aspartate transaminase, lactic dehydrogenase, and C-reactive protein at Emergency department presentation, and hospitalisation were included in RT 1 analysis. PaO_2_/FiO_2_ was available for 155 patients. BMI was available for 160 patients. Pts, patients. PaO_2_/FiO_2_, ratio of arterial oxygen partial pressure to fractional inspired oxygen. BMI, body mass index.

RT analysis identified three variables, namely PaO_2_/FiO_2_ and BMI at ED presentation, and age, that robustly classified patients into risk groups for the *need of follow-up* after discharge, and indicated the cut-offs that maximized the separation among the resulting patient clusters (RT 1, Fig 3). The three groups were: *low probability of need* (PaO_2_/FiO_2_ ≥324 and age <63 years), *intermediate probability of need* (PaO_2_/FiO_2_ <324 and BMI lower than 33 Kg/m^2^ or PaO_2_/FiO_2_ ≥324 and age ≥63 years), and *high probability of need* (PaO_2_/FiO_2_ <324 and BMI ≥33 Kg/m^2^). The ROC_AUC_ for RT 1 was 0.85. Most patients in the *low probability of need* group (65.5%) were discharged from the ED. The fraction of patients that had been hospitalised was higher in the other groups, reaching the totality of patients in the *high probability of need* group.

**Fig 4:**
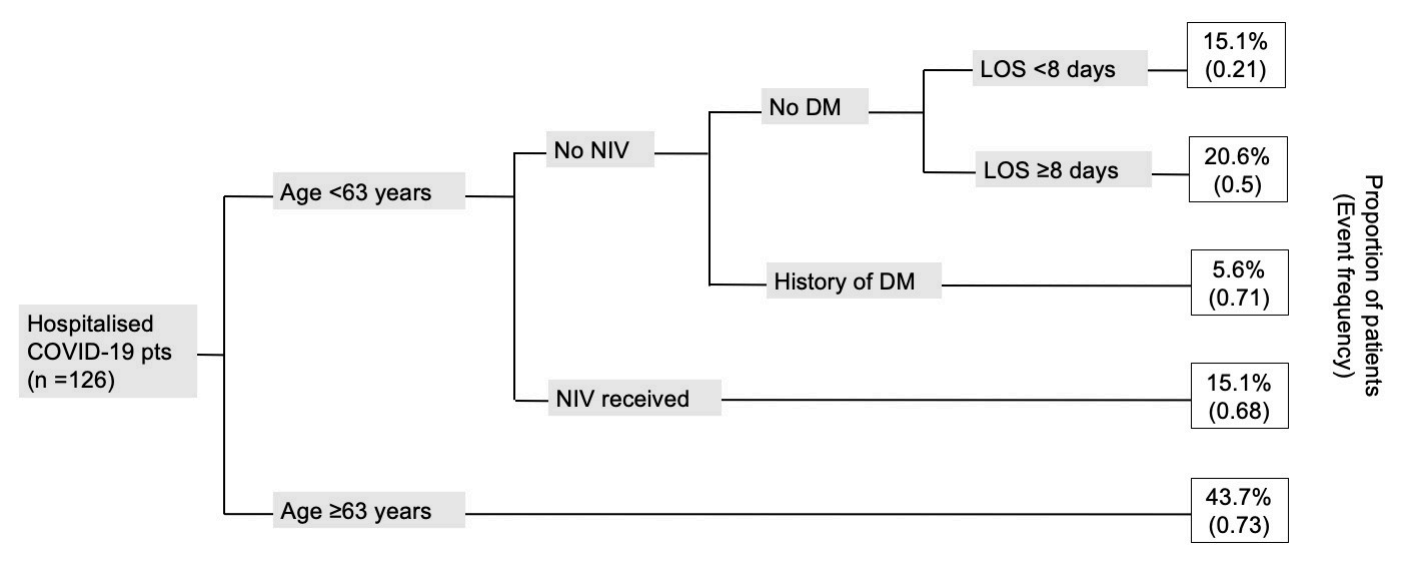
Regression tree analysis (RT 2) to predict the need of post-discharge follow-up among hospitalised patients recovered from COVID-19. Event frequency defines the proportion of patients needing follow-up. Age, gender, ethnicity, history of hypertension, coronary artery disease, chronic kidney disease, diabetes mellitus, administration of non-invasive ventilation, length of stay, and transfer to intensive care unit were included in RT 2 analysis. Pts, patients. NIV, non-invasive ventilation. LoS, length of stay.

When RT analysis was restricted to hospitalised patients (n = 126), four variables emerged as strong predictors of the *need of follow-up* (RT 2, Fig 4). Age, NIV administration, history of DM, and LoS stratified patients into three groups: *low probability of need* (age <63 years, no NIV administration, no history of DM and LoS <8 days), *intermediate probability of need* (age <63 years, no NIV administration, no history of DM and LoS ≥8 days), and *high probability of need* (age <63 years plus NIV or history of DM, or age ≥63 years). The ROC_AUC_ for RT 2 was 0.69.

### PTSD

Decreasing age, female gender and positive psychiatric history were significantly associated with the risk of developing PTSD after COVID-19. Hospitalisation, instead, emerged as protective (Table 5). At multivariable analysis, only female gender and hospitalisation survived as independent predictors of PTSD occurrence. No significant impact was observed for BMI or other comorbidities on PTSD development (Table 5).

**Table 5:** Univariable and multivariable logistic regression analyses predicting PTSD within the entire cohort (n = 185).

	Univariate			Multivariate		
	OR	95% CI	P	OR	95% CI	P
**Age (years)**	0.97	0.94 to 0.99	0.041	0.98	0.95 to 1.02	0.39
**Female gender**	4.88	2.29 to 10.78	0.0001	4.03	1.76 to 9.47	0.0011
**BMI (Kg/m**^**2**^**)**	1.035	0.96 to 1.12	0.38			
***Comorbidities***						
** HTN**	0.48	0.20 to 1.05	0.075			
** CAD**	1.30	0.26 to 5.19	0.72			
** DM**	1.82	0.57 to 5.45	0.29			
** Psychiatric disorder**	2.34	1.04 to 5.26	0.039	2.14	0.86 to 5.28	0.098
**Hospitalisation**	0.25	0.11 to 0.53	0.0004	0.37	0.14 to 0.92	0.033
**ARDS**	0.49	0.21 to 1.10	0.09			

Abbreviations: PTSD, post-traumatic stress disorder; OR, odds ratio; CI, confidence interval; BMI, body mass index; HTN, arterial hypertension; CAD, coronary artery disease; DM, diabetes mellitus; ARDS, acute respiratory distress syndrome.

## Discussion

Here, we present an early analysis of a multidisciplinary follow-up of patients recovered from COVID-19. Between 7 April and 7 May, 2020, 185 patients previously referred to our Institution for COVID-19 were evaluated. Patient characteristics at disease onset were faithful to previously described data [26–28].

Our analysis reveals that many patients, despite apparent clinical recovery at discharge, had clinically relevant medical problems when evaluated after approximately 3 to 4 weeks. For example, one third of them complained of dyspnoea, and 22.2% had a RR >20 breaths/min. Radiological signs of interstitial pneumonia have been described in COVID-19 [29]. Whether these alterations will persist remains to be established. Indeed, viral eradication does not preclude progression to parenchymal fibrosis, and data on pulmonary function after clinical recovery are urgently needed. Uncontrolled HTN was also highly prevalent in our cohort. This is consistent with the hypothesis that SARS-CoV-2 infection may be associated with chronic cardiovascular damage [30], and highlights the need of cardiovascular care in the management of COVID-19 patients.

As high as 68.3% of hospitalised patients were malnourished or at risk of malnutrition, as were 51.0% of patients managed at home. Malnutrition has been reported in hospitalised COVID-19 patients [31], and is likely due to systemic inflammation-related hypercatabolism [32]. ARDS survivors lose lean body mass during acute illness but gain fat mass in the first year after recovery, which may adversely affect functional outcomes [33]. Nutritional assessment and counselling are crucial to these patients. The finding that even patients managed at home were at risk of malnutrition is novel and warrants further investigation. Gastrointestinal symptoms [34] and smell and taste disturbances [35] associated with SARS-CoV-2 infection are possible mechanisms underlying this phenomenon.

We observed cognitive impairment in a quarter of our patients, despite no history of cognitive disorder. Cognitive *sequelae* of COVID-19 might be due to direct viral pathogenicity or immune-mediated mechanisms [8]. In line with a previous study [36], 22.2% of patients developed PTSD. Independent predictors were female gender, in agreement with the prevalence of the disorder in the general population [37], and hospitalisation, which had a protective effect. This might be due to psychosocial stressors such as lockdown and isolation at home, secluded from caregivers, and to a higher vulnerability to inflammation-induced mood and behavioural changes in women [38]. COVID-19 follow-up cannot be separated from an accurate cognitive and psychological monitoring [39].

To set up a follow-up outpatient clinic in times of emergency may be arduous. Apart from logistic difficulties, careful monitoring programs are energy- and time-consuming, and selection of patients who most likely benefit from follow-up programmes may be necessary. We found that older age is a strong predictor of the need of follow-up in both patients who were hospitalised and those who were discharged from the ED. Through recursive partitioning analysis, we identified a hierarchy of independent predictors able to estimate the odds of requiring follow-up after COVID-19. Accordingly, within the entire patient cohort, in addition to older age, lower PaO_2_/FiO_2_ values at ED presentation and obesity discriminated patients not to be lost at hospital discharge. Among hospitalised patients, priority should be given to patients older than 63 years, or to younger patients receiving NIV or with a history of DM, the latter being a known predictor of severity in viral infections, including COVID-19 [40]. In line with our results, age emerged as being an independent predictor of ARDS development in a previous report on severe acute respiratory syndrome (SARS) patients [41]. Likewise, metabolic syndrome-related conditions including obesity and diabetes were found to increase the risk of developing severe illness in patients with Middle East respiratory syndrome coronavirus (MERS-CoV) infection [42, 43]. Although proving causality may be challenging, our findings reinforce the hypothesis that systemic metabolic derangement may precipitate coronavirus diseases, owing to the need of post-recovery monitoring. Potential mechanisms may include endothelial dysfunction, the proinflammatory state, as well as the dysfunctional innate immune response common to both metabolic and viral disorders [43–46].

A main limitation of our study is that instrumental exams were not included in patient monitoring. Nevertheless, clinical measures may be informative surrogates in times of crisis. Our Follow-up COVID-19 Outpatient Clinic was recently upgraded by adding spirometry, electrocardiography, and lung ultrasound in routine evaluations. Patients will be subsequently evaluated at 3 and 6 months from hospital discharge [47]. Another potential limitation is the lack of external validation of our regression tree models. On the other hand, the inclusion of a well characterized population monitored using uniform standards of care, and with the same healthcare access, minimizes the risk of ascertainment bias. Although information on treatment received during the acute phase was not available for all COVID-19 survivors, treatments in the outpatient setting were quite homogenous, whereas in the inpatient setting treatments other than those specifically used for COVID-19 were driven by illness severity, which in our analysis was accounted for by including variables such as administration of non-invasive ventilation, length of stay, and transfer to intensive care unit.

Our study suggests that COVID-19 may leave behind physical and psychological dysfunctions, whose underestimation may be costly in terms of long-term morbidity and mortality. Multidisciplinary follow-up of these patients is therefore crucial to avoid a second wave of late health problems associated with this pandemic. In this sense, selected patient subgroups should be prioritised.

## Supporting information

S1 Dataset The dataset employed for this manuscript. (XLSX)

## References

[1] https://www.who.int/emergencies/diseases/novel-coronavirus-2019 (last access on the 20^th^ of May).

[2] Ciceri F, Castagna A, Rovere-Querini P, De Cobelli F, Ruggeri A, Galli L, et al Early predictors of clinical outcomes of COVID-19 outbreak in Milan, Italy. Clin Immunol. 2020;217:108509 Epub 2020/06/15. 10.1016/j.clim.2020.108509 32535188 PMC7289745

[3] Das KM, Lee EY, Al Jawder SE, Enani MA, Singh R, Skakni L, et al Acute Middle East Respiratory Syndrome Coronavirus: Temporal Lung Changes Observed on the Chest Radiographs of 55 Patients. AJR Am J Roentgenol. 2015;205(3):W267–74. Epub 2015/06/24. 10.2214/AJR.15.14445 . 26102309

[4] Hui DS, Joynt GM, Wong KT, Gomersall CD, Li TS, Antonio G, et al Impact of severe acute respiratory syndrome (SARS) on pulmonary function, functional capacity and quality of life in a cohort of survivors. Thorax. 2005;60(5):401–9. Epub 2005/04/30. 10.1136/thx.2004.030205 15860716 PMC1758905

[5] Wu X, Dong D, Ma D. Thin-Section Computed Tomography Manifestations During Convalescence and Long-Term Follow-Up of Patients with Severe Acute Respiratory Syndrome (SARS). Med Sci Monit. 2016;22:2793–9. Epub 2016/08/09. 10.12659/msm.896985 27501327 PMC4982531

[6] Zhou X, Li Y, Li T, Zhang W. Follow-up of asymptomatic patients with SARS-CoV-2 infection. Clin Microbiol Infect. 2020;26(7):957–9. Epub 2020/04/03. 10.1016/j.cmi.2020.03.024 32234453 PMC7271011

[7] Mack M. Inflammation and fibrosis. Matrix Biol. 2018;68–69:106–21. Epub 2017/12/03. 10.1016/j.matbio.2017.11.010 . 29196207

[8] Natoli S, Oliveira V, Calabresi P, Maia LF, Pisani A. Does SARS-Cov-2 invade the brain? Translational lessons from animal models. Eur J Neurol. 2020 Epub 2020/04/26. 10.1111/ene.14277 32333487 PMC7267377

[9] Akhmerov A, Marban E. COVID-19 and the Heart. Circ Res. 2020;126(10):1443–55. Epub 2020/04/08. 10.1161/CIRCRESAHA.120.317055 . 32252591

[10] Connors JM, Levy JH. COVID-19 and its implications for thrombosis and anticoagulation. Blood. 2020;135(23):2033–40. Epub 2020/04/28. 10.1182/blood.2020006000 32339221 PMC7273827

[11] STROBE Statement for observational studies. Available at: https://www.strobe-statement.org/fileadmin/Strobe/uploads/checklists/STROBE_checklist_v4_combined.pdf Accessed on 22 May 2020.

[12] Bestall JC, Paul EA, Garrod R, Garnham R, Jones PW, Wedzicha JA. Usefulness of the Medical Research Council (MRC) dyspnoea scale as a measure of disability in patients with chronic obstructive pulmonary disease. Thorax. 1999;54(7):581–6. Epub 1999/06/22. 10.1136/thx.54.7.581 10377201 PMC1745516

[13] Brooks R. EuroQol: the current state of play. Health Policy. 1996;37(1):53–72. Epub 1996/06/06. 10.1016/0168-8510(96)00822-6 . 10158943

[14] Kaiser MJ, Bauer JM, Ramsch C, Uter W, Guigoz Y, Cederholm T, et al Validation of the Mini Nutritional Assessment short-form (MNA-SF): a practical tool for identification of nutritional status. J Nutr Health Aging. 2009;13(9):782–8. Epub 2009/10/09. 10.1007/s12603-009-0214-7 . 19812868 PMC12878690

[15] Holvoet E, Vanden Wyngaert K, Van Craenenbroeck AH, Van Biesen W, Eloot S. The screening score of Mini Nutritional Assessment (MNA) is a useful routine screening tool for malnutrition risk in patients on maintenance dialysis. PLoS One. 2020;15(3):e0229722 Epub 2020/03/05. 10.1371/journal.pone.0229722 32130271 PMC7055863

[16] Joaquin C, Alonso N, Lupon J, de Antonio M, Domingo M, Moliner P, et al Mini Nutritional Assessment Short Form is a morbi-mortality predictor in outpatients with heart failure and mid-range left ventricular ejection fraction. Clin Nutr. 2020. Epub 2020/03/15. 10.1016/j.clnu.2020.02.031 . 32169324

[17] Traub J, Bergheim I, Horvath A, Stadlbauer V. Validation of Malnutrition Screening Tools in Liver Cirrhosis. Nutrients. 2020;12(5). Epub 2020/05/08. 10.3390/nu12051306 32375271 PMC7285209

[18] Carson N, Leach L, Murphy KJ. A re-examination of Montreal Cognitive Assessment (MoCA) cutoff scores. Int J Geriatr Psychiatry. 2018;33(2):379–88. Epub 2017/07/22. 10.1002/gps.4756 . 28731508

[19] World Health Organization Quality of Life (WHOQOL)–BREF questionnaire. Available at https://www.who.int/mental_health/media/en/76.pdf?ua=1 Accessed on 22 May 2020.

[20] Levine DW, Dailey ME, Rockhill B, Tipping D, Naughton MJ, Shumaker SA. Validation of the Women's Health Initiative Insomnia Rating Scale in a multicenter controlled clinical trial. Psychosom Med. 2005;67(1):98–104. Epub 2005/01/28. 10.1097/01.psy.0000151743.58067.f0 . 15673630

[21] Sundin EC, Horowitz MJ. Impact of Event Scale: psychometric properties. Br J Psychiatry. 2002;180:205–9. Epub 2002/03/02. 10.1192/bjp.180.3.205 . 11872511

[22] Tluczek A, Henriques JB, Brown RL. Support for the reliability and validity of a six-item state anxiety scale derived from the State-Trait Anxiety Inventory. J Nurs Meas. 2009;17(1):19–28. Epub 2009/11/12. 10.1891/1061-3749.17.1.19 19902657 PMC2776769

[23] Ferguson ND, Fan E, Camporota L, Antonelli M, Anzueto A, Beale R, et al The Berlin definition of ARDS: an expanded rationale, justification, and supplementary material. Intensive Care Med. 2012;38(10):1573–82. Epub 2012/08/29. 10.1007/s00134-012-2682-1 . 22926653

[24] Strauss R, Ewig S, Richter K, Konig T, Heller G, Bauer TT. The prognostic significance of respiratory rate in patients with pneumonia: a retrospective analysis of data from 705,928 hospitalized patients in Germany from 2010–2012. Dtsch Arztebl Int. 2014;111(29–30):503–8, i-v. Epub 2014/08/22. 10.3238/arztebl.2014.0503 25142073 PMC4150027

[25] Breiman L, Friedman J, Stone CJ. Classification and Regression Trees. First edition Boca Raton, FL: Chapman & Hall/CRC Press (Taylor Francis Group).

[26] Huang C, Wang Y, Li X, Ren L, Zhao J, Hu Y, et al Clinical features of patients infected with 2019 novel coronavirus in Wuhan, China. Lancet. 2020;395(10223):497–506. Epub 2020/01/28. 10.1016/S0140-6736(20)30183-5 31986264 PMC7159299

[27] Wang D, Hu B, Hu C, Zhu F, Liu X, Zhang J, et al Clinical Characteristics of 138 Hospitalized Patients With 2019 Novel Coronavirus-Infected Pneumonia in Wuhan, China. JAMA. 2020 Epub 2020/02/08. 10.1001/jama.2020.1585 32031570 PMC7042881

[28] Xu XW, Wu XX, Jiang XG, Xu KJ, Ying LJ, Ma CL, et al Clinical findings in a group of patients infected with the 2019 novel coronavirus (SARS-Cov-2) outside of Wuhan, China: retrospective case series. BMJ. 2020;368:m606 Epub 2020/02/23. 10.1136/bmj.m606 www.icmje.org/coi_disclosure.pdf and declare: no support from any organisation for the submitted work; no financial relationships with any organisations that might have an interest in the submitted work in the previous three years; no other relationships or activities that could appear to have influenced the submitted work. 32075786 PMC7224340

[29] Spagnolo P, Balestro E, Aliberti S, Cocconcelli E, Biondini D, Casa GD, et al Pulmonary fibrosis secondary to COVID-19: a call to arms? Lancet Respir Med. 2020 Epub 2020/05/19. 10.1016/S2213-2600(20)30222-8 32422177 PMC7228737

[30] Zheng YY, Ma YT, Zhang JY, Xie X. COVID-19 and the cardiovascular system. Nat Rev Cardiol. 2020;17(5):259–60. Epub 2020/03/07. 10.1038/s41569-020-0360-5 32139904 PMC7095524

[31] Li T, Zhang Y, Gong C, Wang J, Liu B, Shi L, et al Prevalence of malnutrition and analysis of related factors in elderly patients with COVID-19 in Wuhan, China. Eur J Clin Nutr. 2020;74(6):871–5. Epub 2020/04/24. 10.1038/s41430-020-0642-3 32322046 PMC7175450

[32] Cederholm T, Jensen GL, Correia M, Gonzalez MC, Fukushima R, Higashiguchi T, et al GLIM criteria for the diagnosis of malnutrition—A consensus report from the global clinical nutrition community. J Cachexia Sarcopenia Muscle. 2019;10(1):207–17. Epub 2019/03/29. 10.1002/jcsm.12383 30920778 PMC6438340

[33] Chan KS, Mourtzakis M, Aronson Friedman L, Dinglas VD, Hough CL, Ely EW, et al Evaluating Muscle Mass in Survivors of Acute Respiratory Distress Syndrome: A 1-Year Multicenter Longitudinal Study. Crit Care Med. 2018;46(8):1238–46. Epub 2018/05/05. 10.1097/CCM.0000000000003183 29727365 PMC6051433

[34] Pan L, Mu M, Yang P, Sun Y, Wang R, Yan J, et al Clinical Characteristics of COVID-19 Patients With Digestive Symptoms in Hubei, China: A Descriptive, Cross-Sectional, Multicenter Study. Am J Gastroenterol. 2020;115(5):766–73. Epub 2020/04/15. 10.14309/ajg.0000000000000620 32287140 PMC7172492

[35] Lechien JR, Chiesa-Estomba CM, De Siati DR, Horoi M, Le Bon SD, Rodriguez A, et al Olfactory and gustatory dysfunctions as a clinical presentation of mild-to-moderate forms of the coronavirus disease (COVID-19): a multicenter European study. Eur Arch Otorhinolaryngol. 2020;277(8):2251–61. Epub 2020/04/08. 10.1007/s00405-020-05965-1 32253535 PMC7134551

[36] Liu N, Zhang F, Wei C, Jia Y, Shang Z, Sun L, et al Prevalence and predictors of PTSS during COVID-19 outbreak in China hardest-hit areas: Gender differences matter. Psychiatry Res. 2020;287:112921 Epub 2020/04/03. 10.1016/j.psychres.2020.112921 32240896 PMC7102622

[37] Christiansen DM, Hansen M. Accounting for sex differences in PTSD: A multi-variable mediation model. Eur J Psychotraumatol. 2015;6:26068 Epub 2015/01/22. 10.3402/ejpt.v6.26068 25604705 PMC4300366

[38] Derry HM, Padin AC, Kuo JL, Hughes S, Kiecolt-Glaser JK. Sex Differences in Depression: Does Inflammation Play a Role? Curr Psychiatry Rep. 2015;17(10):78 Epub 2015/08/15. 10.1007/s11920-015-0618-5 26272539 PMC4869519

[39] Holmes EA, O'Connor RC, Perry VH, Tracey I, Wessely S, Arseneault L, et al Multidisciplinary research priorities for the COVID-19 pandemic: a call for action for mental health science. Lancet Psychiatry. 2020;7(6):547–60. Epub 2020/04/19. 10.1016/S2215-0366(20)30168-1 32304649 PMC7159850

[40] Hussain A, Bhowmik B, do Vale Moreira NC. COVID-19 and diabetes: Knowledge in progress. Diabetes Res Clin Pract. 2020;162:108142 Epub 2020/04/13. 10.1016/j.diabres.2020.108142 32278764 PMC7144611

[41] Peiris JS, Chu CM, Cheng VC, Chan KS, Hung IF, Poon LL, et al Clinical progression and viral load in a community outbreak of coronavirus-associated SARS pneumonia: a prospective study. Lancet. 2003;361(9371):1767–72. Epub 2003/06/05. 10.1016/s0140-6736(03)13412-5 12781535 PMC7112410

[42] Alraddadi BM, Watson JT, Almarashi A, Abedi GR, Turkistani A, Sadran M, et al Risk Factors for Primary Middle East Respiratory Syndrome Coronavirus Illness in Humans, Saudi Arabia, 2014. Emerg Infect Dis. 2016;22(1):49–55. Epub 2015/12/23. 10.3201/eid2201.151340 26692185 PMC4696714

[43] Badawi A, Ryoo SG. Prevalence of comorbidities in the Middle East respiratory syndrome coronavirus (MERS-CoV): a systematic review and meta-analysis. Int J Infect Dis. 2016;49:129–33. Epub 2016/06/30. 10.1016/j.ijid.2016.06.015 27352628 PMC7110556

[44] Ciceri F, Beretta L, Scandroglio AM, Colombo S, Landoni G, Ruggeri A, et al Microvascular COVID-19 lung vessels obstructive thromboinflammatory syndrome (MicroCLOTS): an atypical acute respiratory distress syndrome working hypothesis. Crit Care Resusc. 2020;22(2):95–7. Epub 2020/04/17. . 32294809 10.51893/2020.2.pov2 PMC10692450

[45] Htun NS, Odermatt P, Eze IC, Boillat-Blanco N, D'Acremont V, Probst-Hensch N. Is diabetes a risk factor for a severe clinical presentation of dengue?—review and meta-analysis. PLoS Negl Trop Dis. 2015;9(4):e0003741 Epub 2015/04/25. 10.1371/journal.pntd.0003741 25909658 PMC4409149

[46] Odegaard JI, Chawla A. Connecting type 1 and type 2 diabetes through innate immunity. Cold Spring Harb Perspect Med. 2012;2(3):a007724 Epub 2012/03/07. 10.1101/cshperspect.a007724 22393536 PMC3282495

[47] Rovere Querini P, De Lorenzo R, Conte C, Brioni E, Lanzani C, Yacoub MR, et al Post-COVID-19 follow-up clinic: depicting chronicity of a new disease. Acta Biomed. 2020;91(9-S):22–8. Epub 2020/07/24. 10.23750/abm.v91i9-S.10146 . 32701913 PMC8023087

